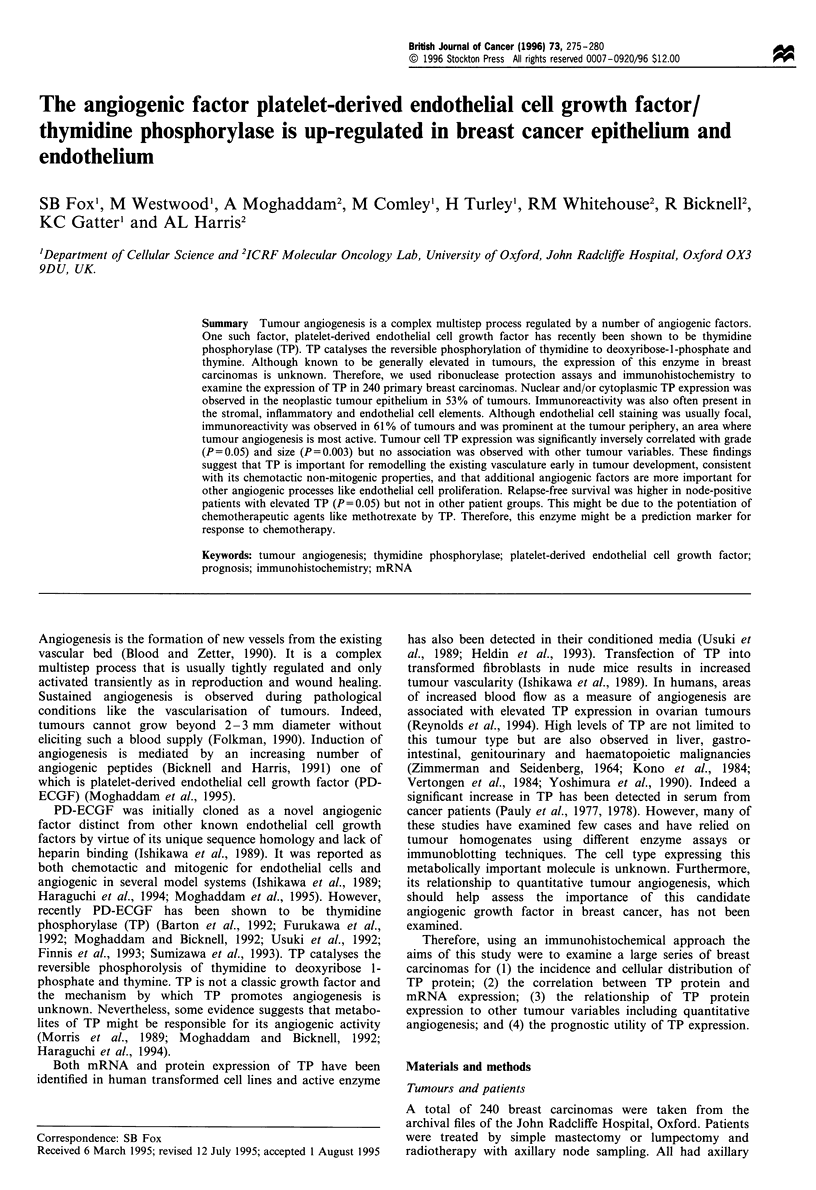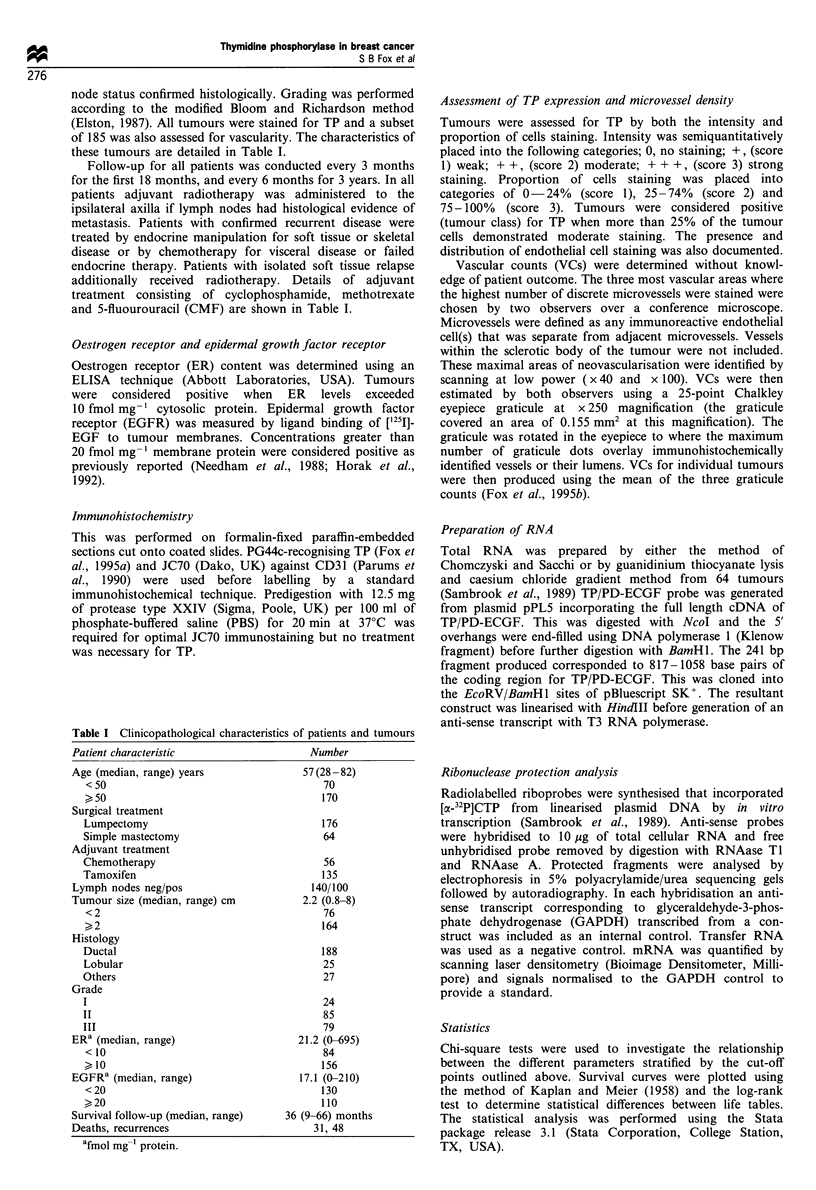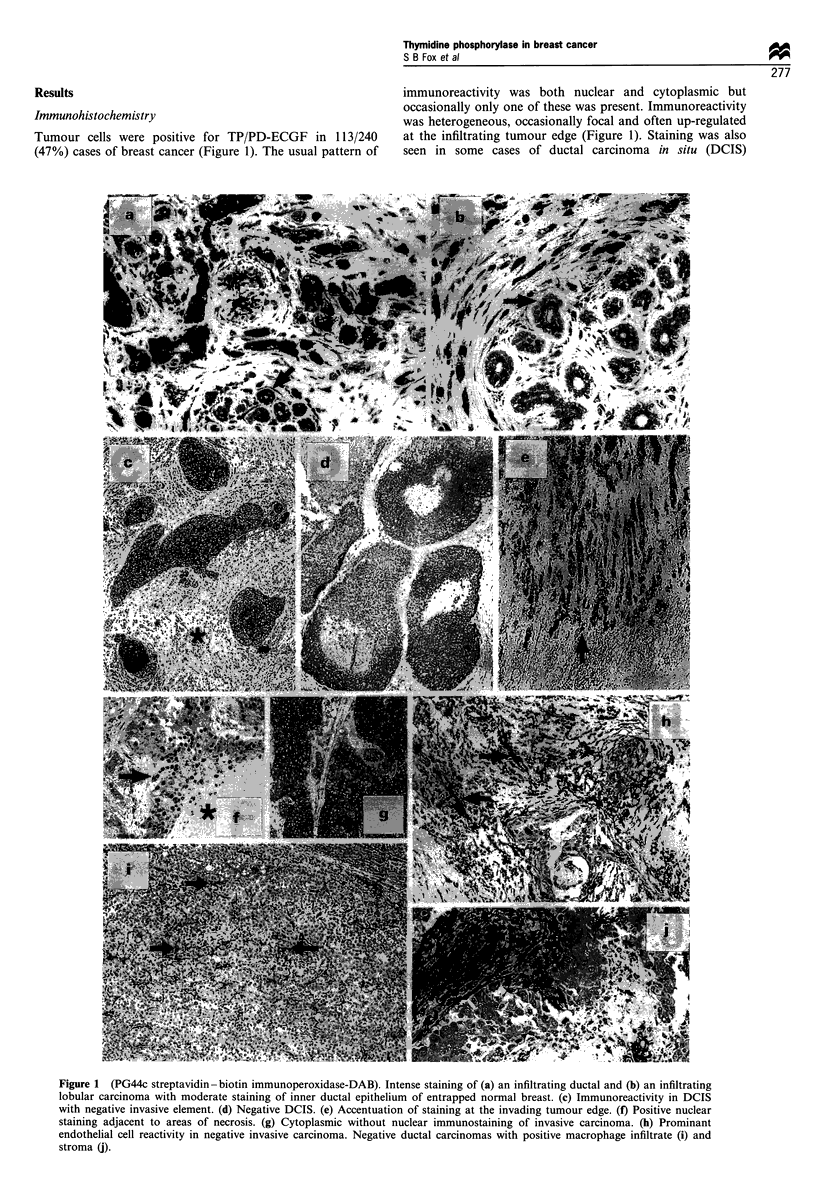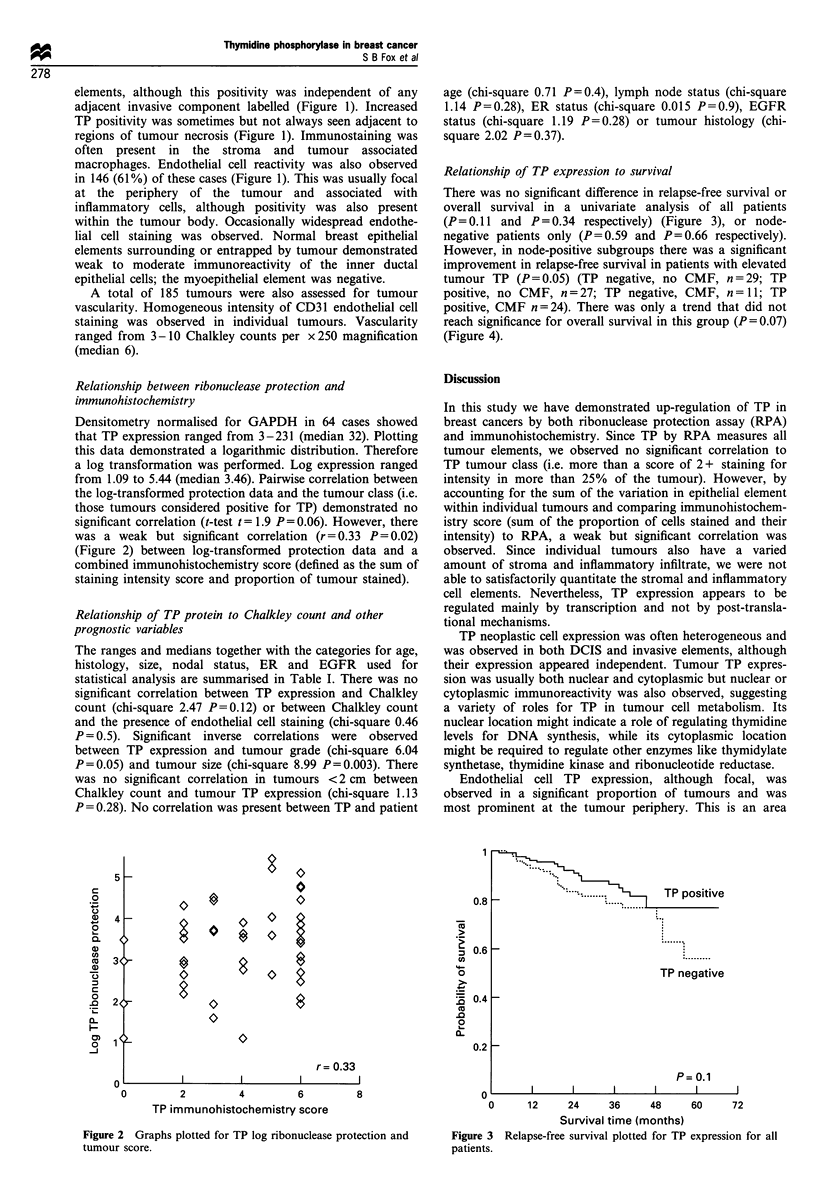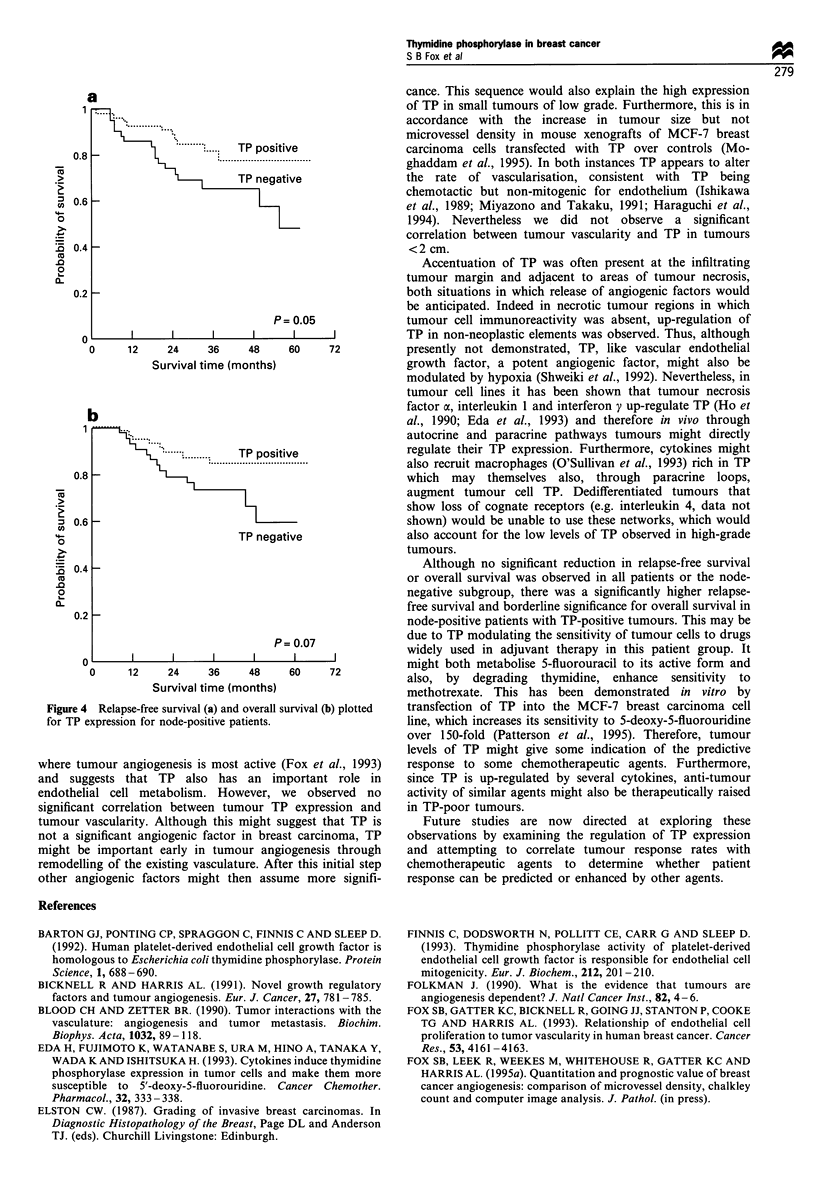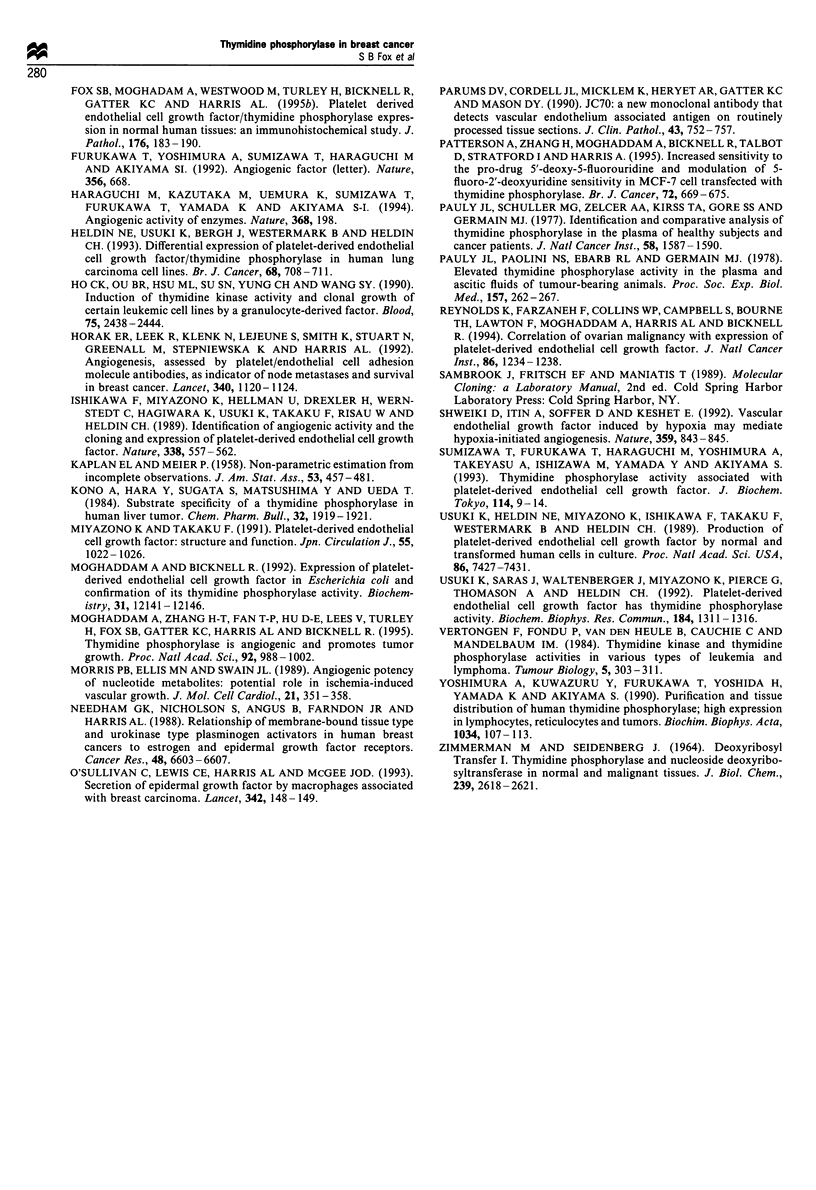# The angiogenic factor platelet-derived endothelial cell growth factor/thymidine phosphorylase is up-regulated in breast cancer epithelium and endothelium.

**DOI:** 10.1038/bjc.1996.49

**Published:** 1996-02

**Authors:** S. B. Fox, M. Westwood, A. Moghaddam, M. Comley, H. Turley, R. M. Whitehouse, R. Bicknell, K. C. Gatter, A. L. Harris

**Affiliations:** Department of Cellular Science, University of Oxford, John Radcliffe Hospital, UK.

## Abstract

**Images:**


British Journal of Cancer (1996) 73, 275-280
© 1996 Stockton Press All rights reserved 0007-0920/96 $12.00

### The angiogenic factor platelet-derived endothelial cell growth factor/ thymidine phosphorylase is up-regulated in breast cancer epithelium and endothelium

SB Fox[1], M Westwood[1], A Moghaddam[2], M Comley[1], H Turley[1], RM Whitehouse[2], R Bicknell[2], KC Gatter[1] and AL Harris[2]

[1]*Department of Cellular Science and* [2]*ICRF Molecular Oncology Lab, University of Oxford, John Radcliffe Hospital, Oxford OX3 9DU, UK.*

**Summary** Tumour angiogenesis is a complex multistep process regulated by a number of angiogenic factors. One such factor, platelet-derived endothelial cell growth factor has recently been shown to be thymidine phosphorylase (TP). TP catalyses the reversible phosphorylation of thymidine to deoxyribose-1-phosphate and thymine. Although known to be generally elevated in tumours, the expression of this enzyme in breast carcinomas is unknown. Therefore, we used ribonuclease protection assays and immunohistochemistry to examine the expression of TP in 240 primary breast carcinomas. Nuclear and/or cytoplasmic TP expression was observed in the neoplastic tumour epithelium in 53% of tumours. Immunoreactivity was also often present in the stromal, inflammatory and endothelial cell elements. Although endothelial cell staining was usually focal, immunoreactivity was observed in 61% of tumours and was prominent at the tumour periphery, an area where tumour angiogenesis is most active. Tumour cell TP expression was significantly inversely correlated with grade ($P=0.05$) and size ($P=0.003$) but no association was observed with other tumour variables. These findings suggest that TP is important for remodelling the existing vasculature early in tumour development, consistent with its chemotactic non-mitogenic properties, and that additional angiogenic factors are more important for other angiogenic processes like endothelial cell proliferation. Relapse-free survival was higher in node-positive patients with elevated TP ($P=0.05$) but not in other patient groups. This might be due to the potentiation of chemotherapeutic agents like methotrexate by TP. Therefore, this enzyme might be a prediction marker for response to chemotherapy.

**Keywords:** tumour angiogenesis; thymidine phosphorylase; platelet-derived endothelial cell growth factor; prognosis; immunohistochemistry; mRNA

Angiogenesis is the formation of new vessels from the existing vascular bed (Blood and Zetter, 1990). It is a complex multistep process that is usually tightly regulated and only activated transiently as in reproduction and wound healing. Sustained angiogenesis is observed during pathological conditions like the vascularisation of tumours. Indeed, tumours cannot grow beyond 2–3 mm diameter without eliciting such a blood supply (Folkman, 1990). Induction of angiogenesis is mediated by an increasing number of angiogenic peptides (Bicknell and Harris, 1991) one of which is platelet-derived endothelial cell growth factor (PD-ECGF) (Moghaddam *et al.*, 1995).

PD-ECGF was initially cloned as a novel angiogenic factor distinct from other known endothelial cell growth factors by virtue of its unique sequence homology and lack of heparin binding (Ishikawa *et al.*, 1989). It was reported as both chemotactic and mitogenic for endothelial cells and angiogenic in several model systems (Ishikawa *et al.*, 1989; Haraguchi *et al.*, 1994; Moghaddam *et al.*, 1995). However, recently PD-ECGF has been shown to be thymidine phosphorylase (TP) (Barton *et al.*, 1992; Furukawa *et al.*, 1992; Moghaddam and Bicknell, 1992; Usuki *et al.*, 1992; Finnis *et al.*, 1993; Sumizawa *et al.*, 1993). TP catalyses the reversible phosphorolysis of thymidine to deoxyribose 1-phosphate and thymine. TP is not a classic growth factor and the mechanism by which TP promotes angiogenesis is unknown. Nevertheless, some evidence suggests that metabolites of TP might be responsible for its angiogenic activity (Morris *et al.*, 1989; Moghaddam and Bicknell, 1992; Haraguchi *et al.*, 1994).

Both mRNA and protein expression of TP have been identified in human transformed cell lines and active enzyme has also been detected in their conditioned media (Usuki *et al.*, 1989; Heldin *et al.*, 1993). Transfection of TP into transformed fibroblasts in nude mice results in increased tumour vascularity (Ishikawa *et al.*, 1989). In humans, areas of increased blood flow as a measure of angiogenesis are associated with elevated TP expression in ovarian tumours (Reynolds *et al.*, 1994). High levels of TP are not limited to this tumour type but are also observed in liver, gastro-intestinal, genitourinary and haematopoietic malignancies (Zimmerman and Seidenberg, 1964; Kono *et al.*, 1984; Vertongen *et al.*, 1984; Yoshimura *et al.*, 1990). Indeed a significant increase in TP has been detected in serum from cancer patients (Pauly *et al.*, 1977, 1978). However, many of these studies have examined few cases and have relied on tumour homogenates using different enzyme assays or immunoblotting techniques. The cell type expressing this metabolically important molecule is unknown. Furthermore, its relationship to quantitative tumour angiogenesis, which should help assess the importance of this candidate angiogenic growth factor in breast cancer, has not been examined.

Therefore, using an immunohistochemical approach the aims of this study were to examine a large series of breast carcinomas for (1) the incidence and cellular distribution of TP protein; (2) the correlation between TP protein and mRNA expression; (3) the relationship of TP protein expression to other tumour variables including quantitative angiogenesis; and (4) the prognostic utility of TP expression.

### Materials and methods

### *Tumours and patients*

A total of 240 breast carcinomas were taken from the archival files of the John Radcliffe Hospital, Oxford. Patients were treated by simple mastectomy or lumpectomy and radiotherapy with axillary node sampling. All had axillary

Correspondence: SB Fox
Received 6 March 1995; revised 12 July 1995; accepted 1 August 1995

node status confirmed histologically. Grading was performed according to the modified Bloom and Richardson method (Elston, 1987). All tumours were stained for TP and a subset of 185 was also assessed for vascularity. The characteristics of these tumours are detailed in Table I.

Follow-up for all patients was conducted every 3 months for the first 18 months, and every 6 months for 3 years. In all patients adjuvant radiotherapy was administered to the ipsilateral axilla if lymph nodes had histological evidence of metastasis. Patients with confirmed recurrent disease were treated by endocrine manipulation for soft tissue or skeletal disease or by chemotherapy for visceral disease or failed endocrine therapy. Patients with isolated soft tissue relapse additionally received radiotherapy. Details of adjuvant treatment consisting of cyclophosphamide, methotrexate and 5-fluourouracil (CMF) are shown in Table I.

*Oestrogen receptor and epidermal growth factor receptor*

Oestrogen receptor (ER) content was determined using an ELISA technique (Abbott Laboratories, USA). Tumours were considered positive when ER levels exceeded 10 fmol $mg^{-1}$ cytosolic protein. Epidermal growth factor receptor (EGFR) was measured by ligand binding of [$^{125}$I]-EGF to tumour membranes. Concentrations greater than 20 fmol $mg^{-1}$ membrane protein were considered positive as previously reported (Needham *et al.*, 1988; Horak *et al.*, 1992).

*Immunohistochemistry*

This was performed on formalin-fixed paraffin-embedded sections cut onto coated slides. PG44c-recognising TP (Fox *et al.*, 1995*a*) and JC70 (Dako, UK) against CD31 (Parums *et al.*, 1990) were used before labelling by a standard immunohistochemical technique. Predigestion with 12.5 mg of protease type XXIV (Sigma, Poole, UK) per 100 ml of phosphate-buffered saline (PBS) for 20 min at 37°C was required for optimal JC70 immunostaining but no treatment was necessary for TP.

**Table I** Clinicopathological characteristics of patients and tumours

| *Patient characteristic* | *Number* |
|---|---|
| Age (median, range) years | 57 (28–82) |
| <50 | 70 |
| ⩾50 | 170 |
| Surgical treatment | |
| Lumpectomy | 176 |
| Simple mastectomy | 64 |
| Adjuvant treatment | |
| Chemotherapy | 56 |
| Tamoxifen | 135 |
| Lymph nodes neg/pos | 140/100 |
| Tumour size (median, range) cm | 2.2 (0.8–8) |
| <2 | 76 |
| ⩾2 | 164 |
| Histology | |
| Ductal | 188 |
| Lobular | 25 |
| Others | 27 |
| Grade | |
| I | 24 |
| II | 85 |
| III | 79 |
| ER[a] (median, range) | 21.2 (0–695) |
| <10 | 84 |
| ⩾10 | 156 |
| EGFR[a] (median, range) | 17.1 (0–210) |
| <20 | 130 |
| ⩾20 | 110 |
| Survival follow-up (median, range) | 36 (9–66) months |
| Deaths, recurrences | 31, 48 |

[a]fmol $mg^{-1}$ protein.

*Assessment of TP expression and microvessel density*

Tumours were assessed for TP by both the intensity and proportion of cells staining. Intensity was semiquantitatively placed into the following categories; 0, no staining; +, (score 1) weak; + +, (score 2) moderate; + + +, (score 3) strong staining. Proportion of cells staining was placed into categories of 0—24% (score 1), 25–74% (score 2) and 75–100% (score 3). Tumours were considered positive (tumour class) for TP when more than 25% of the tumour cells demonstrated moderate staining. The presence and distribution of endothelial cell staining was also documented.

Vascular counts (VCs) were determined without knowledge of patient outcome. The three most vascular areas where the highest number of discrete microvessels were stained were chosen by two observers over a conference microscope. Microvessels were defined as any immunoreactive endothelial cell(s) that was separate from adjacent microvessels. Vessels within the sclerotic body of the tumour were not included. These maximal areas of neovascularisation were identified by scanning at low power (×40 and ×100). VCs were then estimated by both observers using a 25-point Chalkley eyepiece graticule at ×250 magnification (the graticule covered an area of 0.155 $mm^2$ at this magnification). The graticule was rotated in the eyepiece to where the maximum number of graticule dots overlay immunohistochemically identified vessels or their lumens. VCs for individual tumours were then produced using the mean of the three graticule counts (Fox *et al.*, 1995*b*).

*Preparation of RNA*

Total RNA was prepared by either the method of Chomczyski and Sacchi or by guanidinium thiocyanate lysis and caesium chloride gradient method from 64 tumours (Sambrook *et al.*, 1989) TP/PD-ECGF probe was generated from plasmid pPL5 incorporating the full length cDNA of TP/PD-ECGF. This was digested with *Nco*I and the 5′ overhangs were end-filled using DNA polymerase 1 (Klenow fragment) before further digestion with *Bam*H1. The 241 bp fragment produced corresponded to 817–1058 base pairs of the coding region for TP/PD-ECGF. This was cloned into the *Eco*RV/*Bam*H1 sites of pBluescript $SK^+$. The resultant construct was linearised with *Hind*III before generation of an anti-sense transcript with T3 RNA polymerase.

*Ribonuclease protection analysis*

Radiolabelled riboprobes were synthesised that incorporated [α-$^{32}$P]CTP from linearised plasmid DNA by *in vitro* transcription (Sambrook *et al.*, 1989). Anti-sense probes were hybridised to 10 μg of total cellular RNA and free unhybridised probe removed by digestion with RNAase T1 and RNAase A. Protected fragments were analysed by electrophoresis in 5% polyacrylamide/urea sequencing gels followed by autoradiography. In each hybridisation an anti-sense transcript corresponding to glyceraldehyde-3-phosphate dehydrogenase (GAPDH) transcribed from a construct was included as an internal control. Transfer RNA was used as a negative control. mRNA was quantified by scanning laser densitometry (Bioimage Densitometer, Millipore) and signals normalised to the GAPDH control to provide a standard.

*Statistics*

Chi-square tests were used to investigate the relationship between the different parameters stratified by the cut-off points outlined above. Survival curves were plotted using the method of Kaplan and Meier (1958) and the log-rank test to determine statistical differences between life tables. The statistical analysis was performed using the Stata package release 3.1 (Stata Corporation, College Station, TX, USA).

### Results

### *Immunohistochemistry*

Tumour cells were positive for TP/PD-ECGF in 113/240 (47%) cases of breast cancer (Figure 1). The usual pattern of immunoreactivity was both nuclear and cytoplasmic but occasionally only one of these was present. Immunoreactivity was heterogeneous, occasionally focal and often up-regulated at the infiltrating tumour edge (Figure 1). Staining was also seen in some cases of ductal carcinoma *in situ* (DCIS)

**Figure 1** (PG44c streptavidin–biotin immunoperoxidase-DAB). Intense staining of (**a**) an infiltrating ductal and (**b**) an infiltrating lobular carcinoma with moderate staining of inner ductal epithelium of entrapped normal breast. (**c**) Immunoreactivity in DCIS with negative invasive element. (**d**) Negative DCIS. (**e**) Accentuation of staining at the invading tumour edge. (**f**) Positive nuclear staining adjacent to areas of necrosis. (**g**) Cytoplasmic without nuclear immunostaining of invasive carcinoma. (**h**) Prominant endothelial cell reactivity in negative invasive carcinoma. Negative ductal carcinomas with positive macrophage infiltrate (**i**) and stroma (**j**).

elements, although this positivity was independent of any adjacent invasive component labelled (Figure 1). Increased TP positivity was sometimes but not always seen adjacent to regions of tumour necrosis (Figure 1). Immunostaining was often present in the stroma and tumour associated macrophages. Endothelial cell reactivity was also observed in 146 (61%) of these cases (Figure 1). This was usually focal at the periphery of the tumour and associated with inflammatory cells, although positivity was also present within the tumour body. Occasionally widespread endothelial cell staining was observed. Normal breast epithelial elements surrounding or entrapped by tumour demonstrated weak to moderate immunoreactivity of the inner ductal epithelial cells; the myoepithelial element was negative.

A total of 185 tumours were also assessed for tumour vascularity. Homogeneous intensity of CD31 endothelial cell staining was observed in individual tumours. Vascularity ranged from 3–10 Chalkley counts per ×250 magnification (median 6).

### *Relationship between ribonuclease protection and immunohistochemistry*

Densitometry normalised for GAPDH in 64 cases showed that TP expression ranged from 3–231 (median 32). Plotting this data demonstrated a logarithmic distribution. Therefore a log transformation was performed. Log expression ranged from 1.09 to 5.44 (median 3.46). Pairwise correlation between the log-transformed protection data and the tumour class (i.e. those tumours considered positive for TP) demonstrated no significant correlation ($t$-test $t=1.9$ $P=0.06$). However, there was a weak but significant correlation ($r=0.33$ $P=0.02$) (Figure 2) between log-transformed protection data and a combined immunohistochemistry score (defined as the sum of staining intensity score and proportion of tumour stained).

### *Relationship of TP protein to Chalkley count and other prognostic variables*

The ranges and medians together with the categories for age, histology, size, nodal status, ER and EGFR used for statistical analysis are summarised in Table I. There was no significant correlation between TP expression and Chalkley count (chi-square 2.47 $P=0.12$) or between Chalkley count and the presence of endothelial cell staining (chi-square 0.46 $P=0.5$). Significant inverse correlations were observed between TP expression and tumour grade (chi-square 6.04 $P=0.05$) and tumour size (chi-square 8.99 $P=0.003$). There was no significant correlation in tumours <2 cm between Chalkley count and tumour TP expression (chi-square 1.13 $P=0.28$). No correlation was present between TP and patient age (chi-square 0.71 $P=0.4$), lymph node status (chi-square 1.14 $P=0.28$), ER status (chi-square 0.015 $P=0.9$), EGFR status (chi-square 1.19 $P=0.28$) or tumour histology (chi-square 2.02 $P=0.37$).

### *Relationship of TP expression to survival*

There was no significant difference in relapse-free survival or overall survival in a univariate analysis of all patients ($P=0.11$ and $P=0.34$ respectively) (Figure 3), or node-negative patients only ($P=0.59$ and $P=0.66$ respectively). However, in node-positive subgroups there was a significant improvement in relapse-free survival in patients with elevated tumour TP ($P=0.05$) (TP negative, no CMF, $n=29$; TP positive, no CMF, $n=27$; TP negative, CMF, $n=11$; TP positive, CMF $n=24$). There was only a trend that did not reach significance for overall survival in this group ($P=0.07$) (Figure 4).

### Discussion

In this study we have demonstrated up-regulation of TP in breast cancers by both ribonuclease protection assay (RPA) and immunohistochemistry. Since TP by RPA measures all tumour elements, we observed no significant correlation to TP tumour class (i.e. more than a score of 2+ staining for intensity in more than 25% of the tumour). However, by accounting for the sum of the variation in epithelial element within individual tumours and comparing immunohistochemistry score (sum of the proportion of cells stained and their intensity) to RPA, a weak but significant correlation was observed. Since individual tumours also have a varied amount of stroma and inflammatory infiltrate, we were not able to satisfactorily quantitate the stromal and inflammatory cell elements. Nevertheless, TP expression appears to be regulated mainly by transcription and not by post-translational mechanisms.

TP neoplastic cell expression was often heterogeneous and was observed in both DCIS and invasive elements, although their expression appeared independent. Tumour TP expression was usually both nuclear and cytoplasmic but nuclear or cytoplasmic immunoreactivity was also observed, suggesting a variety of roles for TP in tumour cell metabolism. Its nuclear location might indicate a role of regulating thymidine levels for DNA synthesis, while its cytoplasmic location might be required to regulate other enzymes like thymidylate synthetase, thymidine kinase and ribonucleotide reductase.

Endothelial cell TP expression, although focal, was observed in a significant proportion of tumours and was most prominent at the tumour periphery. This is an area

**Figure 2** Graphs plotted for TP log ribonuclease protection and tumour score.

**Figure 3** Relapse-free survival plotted for TP expression for all patients.

**Figure 4** Relapse-free survival (**a**) and overall survival (**b**) plotted for TP expression for node-positive patients.

where tumour angiogenesis is most active (Fox *et al.*, 1993) and suggests that TP also has an important role in endothelial cell metabolism. However, we observed no significant correlation between tumour TP expression and tumour vascularity. Although this might suggest that TP is not a significant angiogenic factor in breast carcinoma, TP might be important early in tumour angiogenesis through remodelling of the existing vasculature. After this initial step other angiogenic factors might then assume more significance. This sequence would also explain the high expression of TP in small tumours of low grade. Furthermore, this is in accordance with the increase in tumour size but not microvessel density in mouse xenografts of MCF-7 breast carcinoma cells transfected with TP over controls (Moghaddam *et al.*, 1995). In both instances TP appears to alter the rate of vascularisation, consistent with TP being chemotactic but non-mitogenic for endothelium (Ishikawa *et al.*, 1989; Miyazono and Takaku, 1991; Haraguchi *et al.*, 1994). Nevertheless we did not observe a significant correlation between tumour vascularity and TP in tumours <2 cm.

Accentuation of TP was often present at the infiltrating tumour margin and adjacent to areas of tumour necrosis, both situations in which release of angiogenic factors would be anticipated. Indeed in necrotic tumour regions in which tumour cell immunoreactivity was absent, up-regulation of TP in non-neoplastic elements was observed. Thus, although presently not demonstrated, TP, like vascular endothelial growth factor, a potent angiogenic factor, might also be modulated by hypoxia (Shweiki *et al.*, 1992). Nevertheless, in tumour cell lines it has been shown that tumour necrosis factor $\alpha$, interleukin 1 and interferon $\gamma$ up-regulate TP (Ho *et al.*, 1990; Eda *et al.*, 1993) and therefore *in vivo* through autocrine and paracrine pathways tumours might directly regulate their TP expression. Furthermore, cytokines might also recruit macrophages (O'Sullivan *et al.*, 1993) rich in TP which may themselves also, through paracrine loops, augment tumour cell TP. Dedifferentiated tumours that show loss of cognate receptors (e.g. interleukin 4, data not shown) would be unable to use these networks, which would also account for the low levels of TP observed in high-grade tumours.

Although no significant reduction in relapse-free survival or overall survival was observed in all patients or the node-negative subgroup, there was a significantly higher relapse-free survival and borderline significance for overall survival in node-positive patients with TP-positive tumours. This may be due to TP modulating the sensitivity of tumour cells to drugs widely used in adjuvant therapy in this patient group. It might both metabolise 5-fluorouracil to its active form and also, by degrading thymidine, enhance sensitivity to methotrexate. This has been demonstrated *in vitro* by transfection of TP into the MCF-7 breast carcinoma cell line, which increases its sensitivity to 5-deoxy-5-fluorouridine over 150-fold (Patterson *et al.*, 1995). Therefore, tumour levels of TP might give some indication of the predictive response to some chemotherapeutic agents. Furthermore, since TP is up-regulated by several cytokines, anti-tumour activity of similar agents might also be therapeutically raised in TP-poor tumours.

Future studies are now directed at exploring these observations by examining the regulation of TP expression and attempting to correlate tumour response rates with chemotherapeutic agents to determine whether patient response can be predicted or enhanced by other agents.

### References

BARTON GJ, PONTING CP, SPRAGGON C, FINNIS C AND SLEEP D. (1992). Human platelet-derived endothelial cell growth factor is homologous to *Escherichia coli* thymidine phosphorylase. *Protein Science*, **1**, 688–690.

BICKNELL R AND HARRIS AL. (1991). Novel growth regulatory factors and tumour angiogenesis. *Eur. J. Cancer*, **27**, 781–785.

BLOOD CH AND ZETTER BR. (1990). Tumor interactions with the vasculature: angiogenesis and tumor metastasis. *Biochim. Biophys. Acta*, **1032**, 89–118.

EDA H, FUJIMOTO K, WATANABE S, URA M, HINO A, TANAKA Y, WADA K AND ISHITSUKA H. (1993). Cytokines induce thymidine phosphorylase expression in tumor cells and make them more susceptible to 5′-deoxy-5-fluorouridine. *Cancer Chemother. Pharmacol.*, **32**, 333–338.

ELSTON CW. (1987). Grading of invasive breast carcinomas. In *Diagnostic Histopathology of the Breast*, Page DL and Anderson TJ. (eds). Churchill Livingstone: Edinburgh.

FINNIS C, DODSWORTH N, POLLITT CE, CARR G AND SLEEP D. (1993). Thymidine phosphorylase activity of platelet-derived endothelial cell growth factor is responsible for endothelial cell mitogenicity. *Eur. J. Biochem.*, **212**, 201–210.

FOLKMAN J. (1990). What is the evidence that tumours are angiogenesis dependent? *J. Natl Cancer Inst.*, **82**, 4–6.

FOX SB, GATTER KC, BICKNELL R, GOING JJ, STANTON P, COOKE TG AND HARRIS AL. (1993). Relationship of endothelial cell proliferation to tumor vascularity in human breast cancer. *Cancer Res.*, **53**, 4161–4163.

FOX SB, LEEK R, WEEKES M, WHITEHOUSE R, GATTER KC AND HARRIS AL. (1995*a*). Quantitation and prognostic value of breast cancer angiogenesis: comparison of microvessel density, chalkley count and computer image analysis. *J. Pathol.* (in press).

FOX SB, MOGHADAM A, WESTWOOD M, TURLEY H, BICKNELL R, GATTER KC AND HARRIS AL. (1995*b*). Platelet derived endothelial cell growth factor/thymidine phosphorylase expression in normal human tissues: an immunohistochemical study. *J. Pathol.*, **176**, 183–190.

FURUKAWA T, YOSHIMURA A, SUMIZAWA T, HARAGUCHI M AND AKIYAMA SI. (1992). Angiogenic factor (letter). *Nature*, **356**, 668.

HARAGUCHI M, KAZUTAKA M, UEMURA K, SUMIZAWA T, FURUKAWA T, YAMADA K AND AKIYAMA S-I. (1994). Angiogenic activity of enzymes. *Nature*, **368**, 198.

HELDIN NE, USUKI K, BERGH J, WESTERMARK B AND HELDIN CH. (1993). Differential expression of platelet-derived endothelial cell growth factor/thymidine phosphorylase in human lung carcinoma cell lines. *Br. J. Cancer*, **68**, 708–711.

HO CK, OU BR, HSU ML, SU SN, YUNG CH AND WANG SY. (1990). Induction of thymidine kinase activity and clonal growth of certain leukemic cell lines by a granulocyte-derived factor. *Blood*, **75**, 2438–2444.

HORAK ER, LEEK R, KLENK N, LEJEUNE S, SMITH K, STUART N, GREENALL M, STEPNIEWSKA K AND HARRIS AL. (1992). Angiogenesis, assessed by platelet/endothelial cell adhesion molecule antibodies, as indicator of node metastases and survival in breast cancer. *Lancet*, **340**, 1120–1124.

ISHIKAWA F, MIYAZONO K, HELLMAN U, DREXLER H, WERNSTEDT C, HAGIWARA K, USUKI K, TAKAKU F, RISAU W AND HELDIN CH. (1989). Identification of angiogenic activity and the cloning and expression of platelet-derived endothelial cell growth factor. *Nature*, **338**, 557–562.

KAPLAN EL AND MEIER P. (1958). Non-parametric estimation from incomplete observations. *J. Am. Stat. Ass.*, **53**, 457–481.

KONO A, HARA Y, SUGATA S, MATSUSHIMA Y AND UEDA T. (1984). Substrate specificity of a thymidine phosphorylase in human liver tumor. *Chem. Pharm. Bull.*, **32**, 1919–1921.

MIYAZONO K AND TAKAKU F. (1991). Platelet-derived endothelial cell growth factor: structure and function. *Jpn. Circulation J.*, **55**, 1022–1026.

MOGHADDAM A AND BICKNELL R. (1992). Expression of platelet-derived endothelial cell growth factor in *Escherichia coli* and confirmation of its thymidine phosphorylase activity. *Biochemistry*, **31**, 12141–12146.

MOGHADDAM A, ZHANG H-T, FAN T-P, HU D-E, LEES V, TURLEY H, FOX SB, GATTER KC, HARRIS AL AND BICKNELL R. (1995). Thymidine phosphorylase is angiogenic and promotes tumor growth. *Proc. Natl Acad. Sci.*, **92**, 988–1002.

MORRIS PB, ELLIS MN AND SWAIN JL. (1989). Angiogenic potency of nucleotide metabolites: potential role in ischemia-induced vascular growth. *J. Mol. Cell Cardiol.*, **21**, 351–358.

NEEDHAM GK, NICHOLSON S, ANGUS B, FARNDON JR AND HARRIS AL. (1988). Relationship of membrane-bound tissue type and urokinase type plasminogen activators in human breast cancers to estrogen and epidermal growth factor receptors. *Cancer Res.*, **48**, 6603–6607.

O'SULLIVAN C, LEWIS CE, HARRIS AL AND MCGEE JOD. (1993). Secretion of epidermal growth factor by macrophages associated with breast carcinoma. *Lancet*, **342**, 148–149.

PARUMS DV, CORDELL JL, MICKLEM K, HERYET AR, GATTER KC AND MASON DY. (1990). JC70: a new monoclonal antibody that detects vascular endothelium associated antigen on routinely processed tissue sections. *J. Clin. Pathol.*, **43**, 752–757.

PATTERSON A, ZHANG H, MOGHADDAM A, BICKNELL R, TALBOT D, STRATFORD I AND HARRIS A. (1995). Increased sensitivity to the pro-drug 5′-deoxy-5-fluorouridine and modulation of 5-fluoro-2′-deoxyuridine sensitivity in MCF-7 cell transfected with thymidine phosphorylase. *Br. J. Cancer*, **72**, 669–675.

PAULY JL, SCHULLER MG, ZELCER AA, KIRSS TA, GORE SS AND GERMAIN MJ. (1977). Identification and comparative analysis of thymidine phosphorylase in the plasma of healthy subjects and cancer patients. *J. Natl Cancer Inst.*, **58**, 1587–1590.

PAULY JL, PAOLINI NS, EBARB RL AND GERMAIN MJ. (1978). Elevated thymidine phosphorylase activity in the plasma and ascitic fluids of tumour-bearing animals. *Proc. Soc. Exp. Biol. Med.*, **157**, 262–267.

REYNOLDS K, FARZANEH F, COLLINS WP, CAMPBELL S, BOURNE TH, LAWTON F, MOGHADDAM A, HARRIS AL AND BICKNELL R. (1994). Correlation of ovarian malignancy with expression of platelet-derived endothelial cell growth factor. *J. Natl Cancer Inst.*, **86**, 1234–1238.

SAMBROOK J, FRITSCH EF AND MANIATIS T (1989). *Molecular Cloning: a Laboratory Manual*, 2nd ed. Cold Spring Harbor Laboratory Press: Cold Spring Harbor, NY.

SHWEIKI D, ITIN A, SOFFER D AND KESHET E. (1992). Vascular endothelial growth factor induced by hypoxia may mediate hypoxia-initiated angiogenesis. *Nature*, **359**, 843–845.

SUMIZAWA T, FURUKAWA T, HARAGUCHI M, YOSHIMURA A, TAKEYASU A, ISHIZAWA M, YAMADA Y AND AKIYAMA S. (1993). Thymidine phosphorylase activity associated with platelet-derived endothelial cell growth factor. *J. Biochem. Tokyo*, **114**, 9–14.

USUKI K, HELDIN NE, MIYAZONO K, ISHIKAWA F, TAKAKU F, WESTERMARK B AND HELDIN CH. (1989). Production of platelet-derived endothelial cell growth factor by normal and transformed human cells in culture. *Proc. Natl Acad. Sci. USA*, **86**, 7427–7431.

USUKI K, SARAS J, WALTENBERGER J, MIYAZONO K, PIERCE G, THOMASON A AND HELDIN CH. (1992). Platelet-derived endothelial cell growth factor has thymidine phosphorylase activity. *Biochem. Biophys. Res. Commun.*, **184**, 1311–1316.

VERTONGEN F, FONDU P, VAN DEN HEULE B, CAUCHIE C AND MANDELBAUM IM. (1984). Thymidine kinase and thymidine phosphorylase activities in various types of leukemia and lymphoma. *Tumour Biology*, **5**, 303–311.

YOSHIMURA A, KUWAZURU Y, FURUKAWA T, YOSHIDA H, YAMADA K AND AKIYAMA S. (1990). Purification and tissue distribution of human thymidine phosphorylase; high expression in lymphocytes, reticulocytes and tumors. *Biochim. Biophys. Acta*, **1034**, 107–113.

ZIMMERMAN M AND SEIDENBERG J. (1964). Deoxyribosyl Transfer I. Thymidine phosphorylase and nucleoside deoxyribosyltransferase in normal and malignant tissues. *J. Biol. Chem.*, **239**, 2618–2621.